# Transcultural nursing leadership: A concept analysis

**DOI:** 10.1016/j.ijnsa.2023.100161

**Published:** 2023-11-03

**Authors:** Gisela Teixeira, Paulo Cruchinho, Pedro Lucas, Filomena Gaspar

**Affiliations:** Nursing Research Innovation and Development Centre of Lisbon (CIDNUR), 1600-190 Lisbon, Portugal

**Keywords:** Concept analysis, Cultural diversity, Leadership, Nursing, Transcultural

## Abstract

**Background:**

Globalisation, wars, competitiveness, and technological innovation have increased workplace diversity, requiring leaders to conduct multinational projects, lead people from different cultural backgrounds, and deliver appropriate services that meet the needs of clients from different cultures. Several concepts are interchangeably used to define and describe leadership in culturally-diverse work environments; one such concept is transcultural leadership. Because of nurses’ global migration and care delivery to patients from different cultural backgrounds, this concept is of interest in the nursing field. While some nurses have been identified as transcultural leaders, no definition of transcultural nursing leadership has been found in the literature.

**Objective:**

This study aimed to develop an operational definition of transcultural nursing leadership.

**Design:**

A concept analysis was performed using Walker and Avant's method.

**Methods:**

A literature search was conducted using electronic databases, including CINAHL and MEDLINE, books, and encyclopaedias. Quantitative, qualitative, mixed- design studies and literature reviews; master's and doctoral theses; interviews; and text and opinion papers published in English, Spanish, or Portuguese that focused on leadership in multicultural work environments overall and in nursing in particular were included. To identify differences and similarities between the concepts, a Correspondence Factor Analysis with the support of the software IRaMuTeQ was undertaken.

**Results:**

A total of 45 documents with definitions or descriptions of the concepts was included, of which five were in the nursing area and 40 in other areas of study. The defining attribute of transcultural nursing leadership is guiding the delivery of culturally-congruent care. Its antecedents were culturally-diverse populations, multicultural nursing teams, and the need to prepare healthcare workers, administrators, academics, professors, researchers, and minorities for transcultural nursing. The consequences identified included culturally-congruent care for patients and optimal health outcomes for all populations. The identified attributes, antecedents, and consequences did not cover the complexity of a culturally-diverse nursing work environment. Thus, the antecedents, attributes, and consequences of transcultural leadership identified in the literature were considered and added.

**Conclusions:**

Transcultural nursing leadership is a concept grounded in nursing theory that has significant implications for nursing management, education, research, and policy. This holds great promise for advancing culturally-congruent care, addressing health disparities, and building highly inclusive and productive nursing teams in an increasingly diverse world.

**What is already known about this topic?**

• Cultural diversity requires leaders capable of navigating across cultures.
• Despite the increasing cultural diversity in the nursing work environment, there are no operational definitions of transcultural nursing leadership.

Alt-text: Unlabelled box

**What this paper adds**

• We found that different concepts were used to describe leadership in culturally-diverse work environments.
• Transcultural nursing leadership is a culturally- sensitive transformation journey of adapting behaviours, processes, and products to the cultural needs of nurses and patients, breaking conventional paradigms, and guiding the delivery of culturally-congruent care.

Alt-text: Unlabelled box

## 1 Background

Globalisation, wars, competitiveness, and technological innovation have increased the complexity, ambiguity, and diversity of the workplace. The increasing interconnectedness and interdependence among nations and the exchange of people, goods, services, and information across national borders have contributed to cultural diversity in workplaces, communities, and worldwide. This scenario requires leaders able to conduct multinational projects, to lead people or groups from different cultural backgrounds in organisations, and to deliver appropriate services and create products that meet the needs of their clients from different cultures and with different expectations (Abyad, 2011; Bonsu and Twum-Danso, 2018; Cabrera and Unruh, 2012; Cunha et al., 2007; Derungs, 2010; Frawley and Fasoli, 2012; Handin and Steinwedel, 2006; Hanges et al., 2016; Lakshman, 2013; Sertel et al., 2022; Vilas-Boas and Davel, 2018; Windiarti et al., 2014; Yukl, 2013)

Culture is a variable that must be considered when analysing leadership between a leader and a follower from different countries or cultures and when services or products are being designed for clients of different cultural backgrounds. Culture is defined as a set of values, beliefs, norms, lifestyles, arts, and other human products that are learned, shared, and socially- transmitted between generations, guiding people's thoughts, behaviours, decisions, actions, and worldviews in a standardised manner over time and in different geographical locations (Leininger, 2006a; Purnell, 2005).

Particularly in healthcare organizations, just as culture shapes healthcare workers’ expectations of leadership and influences how each one perceives, identifies, defines, and solves problems in the workplace (Andrews, 2016), it also shapes the expectations and perceptions of quality that patients may have of the products and services provided by the healthcare organisation or unit. Patients’ culture shapes the way they perceive the world, health, illness processes, and care (Leininger and McFarland, 2006). For multicultural organisations to succeed, it is important for teams to effectively improve their job satisfaction, retention, and achievement of common goals and to deliver services and products of quality that satisfy the served patients and communities.

Given the global increase in cultural diversity among nurses and patients in the nursing work environment, transcultural leadership within the nursing domain must be understood, clarified, and defined. Leininger introduced the concept of *transcultural nursing administration* in her work, defining it as the ‘creative and knowledgeable process of assessing, planning, and making decisions and policies that will facilitate educational and clinical service goals that take into account cultural caring values, beliefs, symbols, and lifeways of people of diverse and similar cultures for beneficial outcomes’ (Leininger, 2002, p. 563). Despite the identification of transcultural nurse leaders engaged in this process (Leininger and McFarland, 2002), no definition of transcultural nursing leadership has been found. In addition, several concepts seem to be used interchangeably to define and describe leadership in culturally-diverse work environments, such as cross-cultural leadership and intercultural leadership. This study aimed to explore these concepts and develop an operational definition of transcultural nursing leadership. To the best of our knowledge, this is the first study conducted with this objective.

## 2 Methods

A concept analysis was undertaken utilising the methodology established by Walker and Avant (2019), who stated that it is useful for refining ambiguous terms in theory, education, research, and practice, developing operational definitions with a clear theoretical basis, providing an understanding of the attributes of a concept, facilitating the development of an instrument in research, and assisting in the development of the language of nursing. This is a process that clarifies the meaning of a concept so that it is understood in a similar way by all those who use it in the same context, time, and situation (Madureira et al., 2021). Walker and Avant's method is widely used in nursing because of its ease of understanding and mastery for those starting out in this intellectual exercise.

This concept analysis was performed by following the eight steps of Walker and Avant's (2019) methodology: (1) choose a concept, (2) determine the purpose of analysis, (3) identify all uses of the concept, (4) determine the defining attributes, (5) identify a model case, (6) identify additional cases, (7) identify antecedents and consequences, and (8) define empirical referents.

According to Walker and Avant (2019), a concept related to work that has always caused some degree of unease must be chosen. Above all, it should reflect the topic or area of greatest interest to those developing the conceptual analysis. For this study, the choice of the concept of transcultural nursing leadership arose from the absence of a comprehensive definition of this construct in the nursing domain and the identification of a set of terms in the literature that are apparently used to define and describe the same phenomenon. Therefore, this concept analysis aimed to clarify and delineate these terms and develop and propose an operational definition of transcultural nursing leadership.

### 2.1 Data collection

Two search strategies were designed that included similar concepts identified in the English language in academic and scientific literature: *cross-cultural, transcultural,* and *intercultural*. The first search strategy aimed to comprehensively identify the definitions and characteristics of the concept of interest regardless of the field.

**[Title]** *(cross-cultural* **OR** *transcultural* **OR** *intercultural)* **AND** leader*

The second strategy aimed to find definitions and characteristics of transcultural leadership in nursing:

**[Title]** *(cross-cultural* **OR** *transcultural* **OR** *intercultural)* **AND** leader* **AND** nurs*

Both search strategies were applied in the following databases: Academic Search Complete, B-ON, Business Source Complete, CINAHL, Cochrane Database of Systematic Reviews, MedicLatina, Medline, Nursing & Allied Health Collection, Psychology and Behavioural Sciences Collection, eBook Nursing Collection, ScienceDirect, and Wiley Online Library. All quantitative, qualitative, mixed- design studies, literature reviews, master's and doctoral theses, interviews, and text and opinion papers published in English, Spanish, and Portuguese, without limit in the period of publication, available in full text, and defining the concept or describing its characteristics were considered. According to Walker and Avant (2019), a wide reading of various sources is necessary to identify the many uses of this concept. Thus, dictionaries, encyclopaedias, and books were consulted.

**Fig. 1:**
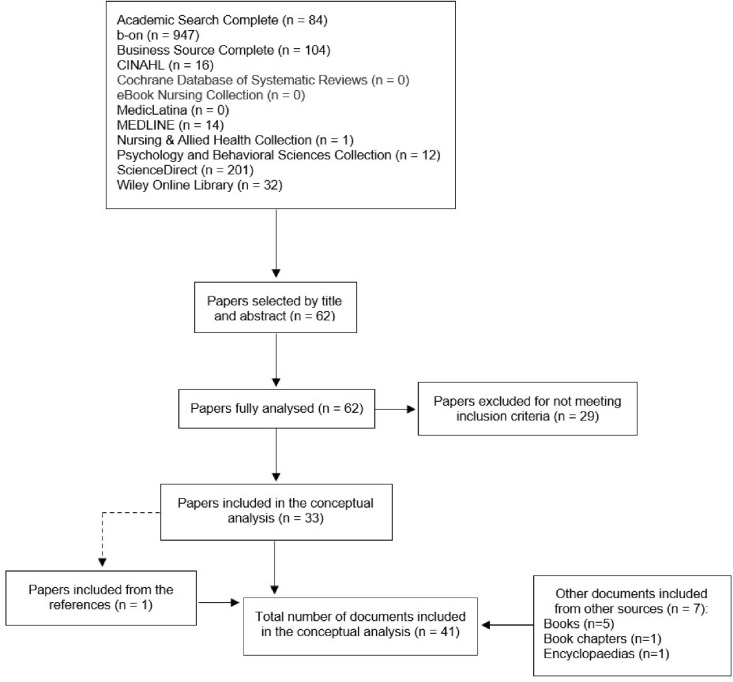
Flow chart of the first study selection process of the concept analysis.

**Fig. 2:**
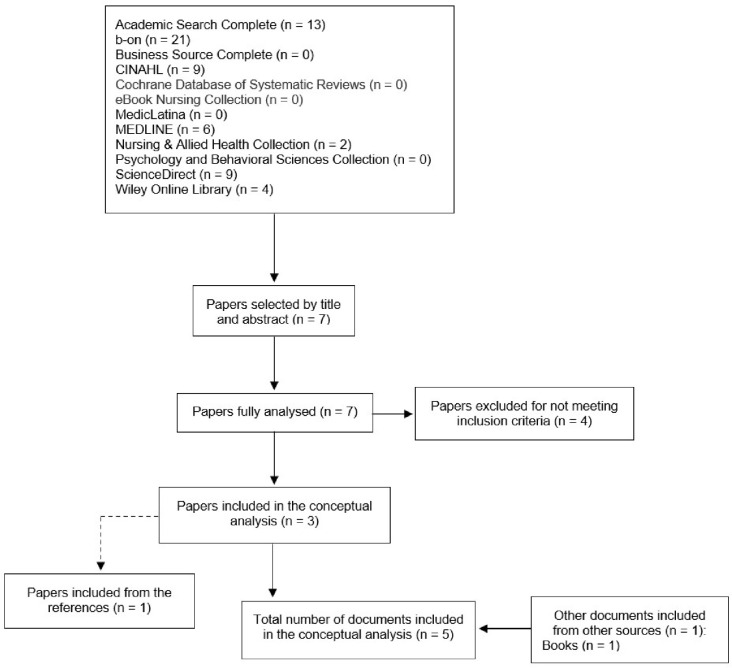
Flow chart of the second study selection process of the concept analysis.

After removing duplicates from both strategies, 45 documents with definitions or descriptions of the concept were included, of which five were in the nursing area and 40 were in other areas of study. The document selection process for both strategies is depicted in Fig. 1, Fig. 2. A fourth concept–global leadership–was identified in the literature and added to the analysis. The definitions and characteristics were organised according to the concept and type of source.

### 2.2 Data analysis

An analysis of the textual data with the support of the Interface de R pour les Analyses Multidimensionnelles de Textes et de Questionnaires (IRaMuTeQ) was undertaken to identify the differences and similarities among the concepts of transcultural leadership, cross-cultural leadership, intercultural leadership, and global leadership*.* This software allows visualisation of the position and structure of words in a text, links, and other textual features, making it possible to detect indicators and intuitively visualise the structure and environments of the text to be analysed (Klamt and Santos, 2021). To process the *corpus* of analysis in the software, it was transformed into a mathematical text *corpus* according to the required guidelines (Camargo and Justo, 2018). The text *corpus* comprised 45 texts, each consisting of descriptions and concept characteristics from one source. The texts were identified from *p_1 to *p_45 and categorised according to the following concepts:

• *Leader _1 (texts using the concept of *cross-cultural leadership*)
• *Leader _2 (text using the concept of *intercultural leadership*)
• *Leader _3 (text using the concept of *transcultural leadership*)
• *Leader _4 (text using *global leadership*)

A Correspondence Factor Analysis (CFA) was performed to analyse whether the active forms present in the four concepts were randomly distributed or whether there was a difference in the forms used to describe their characteristics. This procedure allows the analysis of textual production according to characterisation variables with at least two modalities (Salviati, 2017), enabling the comparison of textual production (Camargo and Justo, 2018) among the four concepts. For this analysis, only active forms (main words; namely, nouns, pronouns, verbs, adverbs, and adjectives) were considered, excluding supplementary forms (definite and indefinite articles, propositions, and conjunctions).

CFA graphically illustrates the data, showing how close or far apart the classes or forms are. The interpretation is based on a graphic representation of the associations between rows and columns. The columns express concepts, whereas the lines correspond to all forms of occurrence. The interpretation of an axis in CFA in a linguistic context is defined by the opposition between extreme points (Bertin and Atanassova, 2015).

## 3 Results

The literature on leadership in culturally-diverse work environments has developed in business (Abyad, 2011; Bird and Mendenhall, 2016; Caligiuri and Tarique, 2012; Foulkes, 1994; Handin and Steinwedel, 2006; Lakshman, 2013; Rao-Nicholson et al., 2020), engineering (Windiarti et al., 2014), and education (Frawley and Fasoli, 2012; Hajisoteriou and Angelides, 2014; Karim, 2003; Morrison, 2012; Walker and Shuangye, 2007). In nursing, no operational definitions have been found, and only brief references have been made to transcultural or global nurse leaders in healthcare environment (Bragadóttir et al., 2020; Douglas et al., 2014; Leininger and McFarland, 2002; Marrone, 2016; Russell, 2022). Of the five documents on nursing included in this concept analysis, only three used the term ‘transcultural’ to describe leadership characteristics (Leininger and McFarland, 2002; Marrone, 2016; Russell, 2022). The remaining two used the terms ‘cross-cultural’ (Douglas et al., 2014) and ‘global’ (Bragadóttir et al., 2020). The concept of transcultural leadership was delimited from the concepts of global leadership and cross-cultural leadership, and partially diluted in the concept of intercultural leadership (Supplementary Material 1).

### 3.1 Uses of the concepts

The common use of ‘cross-cultural leadership’ is to describe a process of influencing, motivating, and enabling a group of people in a culturally-diverse work environment to commit to, accomplish tasks and goals, contribute to the success of an organisation, and identify with the group/organisation and organisational culture (Lakshman, 2013; Windiarti et al., 2014). Cross-cultural leadership enables a process of interaction between managers and employees representing different cultural backgrounds (Suutari et al., 2002), where employees have different values, customs, ethical norms, laws, and traditions shaped by their national or social origins (Bonsu and Twum-Danso, 2018). This process occurs only if there are significant differences between the leader and followers in terms of their knowledge and meaning systems (Akiga and Lowe, 2004). This terminology is widely used to describe the leadership between expatriate managers and local employees (Ersoy, 2014; Festing and Maletzky, 2011; Rao-Nicholson et al., 2020; Scandura and Dorfman, 2004; Suutari et al., 2002; Tsai et al., 2019) and is used in comparative studies or studies that describe leadership attributes in different countries or social cultures (Scandura and Dorfman, 2004). The basic principle of cross-cultural leadership is the influence of cultures on expectations regarding leader-follower interaction and the need for leaders to consider the implications of differences in the knowledge and meaning systems of their followers and to incorporate these differences into the influenced process (Akiga and Lowe, 2004; Suutari et al., 2002). To be effective in this process, leaders need to know and understand how people from different cultures view them and interpret their actions (Yukl, 2013), adjust their leadership behaviours according to the expectations of their followers, adjust the behaviours of their subordinates, or invest in mutual adjustment (Festing and Maletzky, 2011; Tsai, 2022).

Concerning the concept of ‘intercultural leadership’, Walker and Shuangye (2007) simplify it by stating that there is no ‘right way of doing things’ for intercultural leaders; i.e., the way of leading may change according to the cultural context or culture of the employee or client. This implies understanding each culture to determine the behaviour of the client or employee and the ability to anticipate positive change, transmit mission and direction, generate motivation and cooperation, respect diversity and different perspectives, and promote individuality and unity (Abyad, 2011).

The common use of ‘global leadership’ suggests that it is a process aimed at building new types of global alliances and influencing a global workforce with different cultural norms and expectations to adopt a shared vision and work together towards a common goal (Hanges et al., 2016; Morrison, 2012). For example, leading teams or projects involving people from different geographical locations requires navigating the cultural, social, political, technological, and financial differences between countries and regions (Bird and Mendenhall, 2016; Cabrera and Unruh, 2012) and understanding how they can affect global business decisions and relationships (Story, 2011).

Transcultural leadership has been defined as the process of articulating, implementing, and nurturing a global cultural vision and creating multicultural synergy (Foulkes, 1994). According to Smith (2013), a transcultural leader exerts a positive influence on several different groups of people operating within the same organisation or community and uses his or her understanding of culture to create an inclusive environment that transcends the man-made boundaries of culture and facilitates intentional cooperative action. What distinguishes transcultural leadership from the other three concepts is the journey of transformation (Derungs, 2010), which is one of the defining attributes of transcultural leadership identified (Table 1).

**Table 1:** Overall defining attributes of transcultural leadership and in nursing.

Defining attributes	
Overall	In nursing
■ Sensibility to cultural differences and power imbalances (Cunha et al., 2007; Derungs, 2010; Foulkes, 1994; Smith, 2013); ■ Transcultural competence (Derungs, 2010); ■ Adaptation of behaviours, processes and products to the cultural expectations of employees and clients (Cunha et al., 2007; Derungs, 2010); ■ Breaking conventional paradigms (Derungs, 2010); ■ Transformation journey (Derungs, 2010).	■ Guiding the delivery of culturally-congruent care (Leininger and McFarland, 2002; Marrone, 2016; Russell, 2022).

### 3.2 Defining attributes of transcultural leadership

In Walker and Avant's methodology (2019), determining the defining attributes of a concept is the core of conceptual analysis. The effort lies in showing the set of attributes that are most frequently associated with the concept, which enables one to obtain the broadest vision and delimit it from other related concepts. Table 1 presents the defining attributes of transcultural leadership and nursing.

Transcultural leaders act according to the expectations of the organisation, community, competitors, and clients (Cunha et al., 2007; Derungs, 2010). Effectiveness requires leaders to be culturally sensitive by acknowledging and understanding the peculiarities of each culture, the diversity of the values of the people they relate to, acquiring competencies to work with that diversity, be motivated to face it, and manage it harmoniously (Cunha et al., 2007). Transcultural competence is mandatory. According to Derungs (2010), transcultural competence is a lifelong learning process that entails professional training or a place for regular reflection that can be assured only through interactions with others. It embraces personal qualities (awareness of one's own integrity, potential, and limits; recognition of the impact of cultural paradigm on cognitive, affective, and behavioural patterns in interaction and relationship building), interactive qualities (ability to enter into dialogue, go beyond cultural frames in order to understand opposite points of view, and create with others new patterns of understanding, respecting, and valuing as the fundamental basics for dialogue), methodological qualities (ability to apply the principles of transcultural competence in practicing management and leadership), and quality of knowledge (understanding of the dynamics of culture and power; the complexity of causes and consequences at individual, collective, and organizational levels in regards to cognitive, affective, and behavioural dimensions).

Transcultural leadership is greater than the sum of understanding cultures and adapting behaviours to the cultural context and followers’ expectations. It drives a new organisational culture and a new paradigm, and the leader is responsible for managing this transition (Derungs, 2010). It is a process of transformation, a form of approach to prepare and guide people and organisations for and through challenges arising from globalisation, increased competition, and power asymmetries (Derungs, 2010).

Transcultural leaders are not just leaders who know how to manage diversity; they also break conventional paradigms and promote sustainable transformation, bringing a new set of values or a new culture that emerges from diversity (Derungs, 2010). In addition to seeking unity among people from different cultural backgrounds by building positive relationships based on mutual understanding and respect, there is also a process of transformation of the leader, individuals, and community (Derungs, 2010). Thus, the target population involved in this transformation process comprises not only the nurses being led but also the community. Healthcare setting administrators and policymakers are engaged in a process to become culturally aware, knowledgeable, sensible, and proactive to make changes that improve the quality of nurses’ work environment and health outcomes for all populations, considering the needs of people from different cultural backgrounds.

Culturally-congruent care requires nurses to learn how to provide culturally acceptable and appropriate nursing care to diverse populations (Russell, 2022). To lead in the current healthcare environment, Marrone (2016) argued that transcultural nurses must assume positions from which they can influence the development of new knowledge and innovations on local, regional, national, and international boards; guide the vision of nursing and healthcare; and design healthcare delivery systems and environments where transformed healthcare models can be sustained and flourish. This transformation requires the leader's guidance to unlearn paradigms and confront fear in order to progress towards a more adaptive paradigm that contributes to continuous improvement and creates strategic value (Derungs, 2010).

### 3.3 Model case

A model case is an instance of a concept in which all defining attributes are demonstrated (Walker and Avant, 2019).

Johanna is a nurse manager on an inpatient ward of a European hospital serving a culturally-diverse community, mainly from the Middle East. She is from South Africa and leads a team of British, Filipino, and Moroccan nurses. She understands that nurses and patients from different cultures require different approaches.

Johanna acknowledges that cultural values and communication styles may affect nurses’ behaviours and relationships. She knows that high levels of performance orientation are desired in both the Philippines and Morocco and that societies with this cultural value expect direct and explicit communication. Despite this similarity, when Johanna communicates with Moroccan nurses, she uses louder and more expressive speech, which is more common in Arab cultures. When she communicates with Filipino nurses, she uses a softer tone of voice and less expressive speech, favoured by Asian cultures.

Johanna has learned about cultural norms and values in each country from which her staff and patients come. She founded a Committee for Diversity with other nurse managers of the hospital to improve current policies and procedures to be more inclusive and prevent xenophobia and discrimination against patients and healthcare workers.

Johanna has adapted her unit procedures to meet the cultural needs of the patients. In the Middle East, the majority of patients are Muslims, praying five times a day facing Mecca. During the month of Ramadan, they fast from before the first light at dawn to sunset. Johanna converted an empty room into a small prayer room and adjusted mealtimes in the unit for Muslim patients during Ramadan.

Johanna encourages her nursing staff to think creatively and find innovative solutions to problems, even if they go against conventional nursing knowledge. For instance, due to low patient adherence to treatments, it was suggested by the nursing team to ask patients and their relatives to bring Zamzam water and integrate it into nursing care (for example, during hygiene) to build patients’ confidence in nurses and improve their adherence to nurses’ recommendations.

Johanna has implemented training programmes for nursing staff to ensure that they know and understand patients’ cultural norms, beliefs, values, and health practices. She created a patient feedback system that allows her unit to receive feedback on their nursing services and make adjustments where necessary and proposed that the director of nursing hire Arabic speaker interpreters with knowledge of medical terms. She has also implemented diversity and inclusion training programs for her nursing staff to ensure that they understand and respect their colleagues’ cultural backgrounds. She created opportunities for her nursing staff to share cultural traditions and experiences, building a sense of community and understanding among them. Every two months, she provides monthly in-service education about different cultures and plans a monthly social activity to be enjoyed by the nursing staff, such as sharing traditional meals at lunchtime.

Johanna's ability to navigate across cultural differences has been transformative for nurses, the unit, and herself. The nurses have become more aware, sensitive, and knowledgeable about cultural differences and their impact on nursing care and their relationships with patients and colleagues. The work environment became increasingly supportive and inclusive. Johanna has become more effective in culturally-diverse work environments.

### 3.4 Borderline case

A borderline case is an example that contains some but not all of the defining attributes of the concept (Walker and Avant, 2019).

Mark is a nurse manager on an inpatient ward of a European hospital serving a culturally-diverse community, mainly from the Middle East. He leads a nursing team of British, Ghanaian, and Brazilian nurses.

Mark understands that each country has unique cultural expectations and recognises that nurses and patients from different cultures require different approaches. He acknowledges that there may be power imbalances in the nurse-patient relationship due to cultural and language barriers.

Mark has adopted an authoritarian leadership style and provides guidance to all nurses, regardless of their level of professional autonomy. To meet Muslim patients’ religious needs, Mark adapted an empty room into a small prayer room for independent patients but refused to adjust the mealtimes in the unit for Muslim patients during Ramadan, leading some patients to ask for discharge against medical advice because they were unable to fast when they should and eat when they could.

Mark recognises that what works in one culture may not work in another but is not open to new ideas and approaches if they go against scientific evidence. For instance, he does not allow patients or their relatives to bring Zamzam water to be included in the nursing practice because ‘there is no evidence of its benefits’. He advises nurses to enrol in cultural training programs to ensure that they know and understand different cultural norms, beliefs, values, and health practices. However, he does not provide in-service or working hours for nurses to attend these programmes.

### 3.5 Contrary case

A contrary case is a clear instance of what the concept is not.

Claire is a nurse manager on an inpatient ward of a European hospital serving a culturally-diverse community, mainly from the Middle East. She leads a nursing team of British, Filipino, and Indian nurses.

Claire is not keen to learn about cultural differences and does not acknowledge the power imbalances between nurses and patients or its impact on nursing care and teamwork. Her communication style is characterised by verbal directness and loud and expressive speech and is considered rude by Filipino nurses, who feel uncomfortable and avoid meeting Claire when they need to solve problems.

Claire argues that there is no need to adjust or change the unit or auxiliary services to meet the religious needs of Muslim patients because she does not share the same beliefs. One nurse suggested creating a working group to develop in-services about cultural differences in the healthcare team and about culturally-congruent care because nursing staff are unfamiliar with Muslims’ cultural practices. Claire answered that these themes are not a priority and that nurses and patients from other cultural backgrounds would have to adjust to the local culture.

### 3.6 Antecedents and consequences

#### 3.6.1 Antecedents

Antecedents are events that occur or are in place prior to the emergence of a particular concept (Walker and Avant, 2019). In other words, these are the events, circumstances, or conditions that give rise to the concept. Table 2 summarises the antecedents of transcultural leadership, particularly in nursing.

**Table 2:** Antecedents of transcultural leadership.

Antecedents	
Overall	In nursing
■ Globalisation (Derungs, 2010); ■ Cultural diversity in the local and transnational workplace (Cunha et al., 2007; Derungs, 2010; Foulkes, 1994; Harvey, 2015; Smith, 2013); ■ Clients, suppliers, and partners from different cultures (Cunha et al., 2007); ■ Different expectations of the leader and followers from different cultures regarding leadership (Cunha et al., 2007).	■ Culturally-diverse populations (Marrone, 2016; Russell, 2022); ■ Foreign nurses in teams (Leininger and McFarland, 2006) ■ Need to prepare the healthcare workers, administrators, academics, professors, researchers, and minorities for transcultural nursing (Leininger and McFarland, 2002).

These antecedents of transcultural leadership can be divided into three major categories: (a) globalisation, (b) cultural diversity in the workplace and communities, and (c) differences in knowledge and meaning systems.

Globalisation is a consequence of the increasing interconnectedness and interdependence between nations and the exchange of people, goods, services, and information across national borders, which, in turn, requires leaders who can understand and navigate the cultural, social, political, economic, and technological differences between countries and regions (Bonsu and Twum-Danso, 2018; Cabrera and Unruh, 2012; Derungs, 2010; Handin and Steinwedel, 2006; Hanges et al., 2016; Lakshman, 2013; Morrison, 2012; Sertel et al., 2022; Yukl, 2013).

Transcultural leadership occurs when leaders work in a different culture, when they work in their own country but lead multicultural teams or companies whose employees come from different cultural backgrounds, and when they perform activities in which it is necessary to deal with employees, clients, subordinates, superiors, suppliers, and partners from different cultures (Cunha et al., 2007). Cultural diversity in the workplace, communities, and the world at large has led to the need for leaders who can effectively lead people or groups from different cultural backgrounds and produce appropriate responses to the needs of clients with different cultures and expectations (Abyad, 2011; Cunha et al., 2007; Derungs, 2010; Douglas et al., 2014; Frawley and Fasoli, 2012; Hanges et al., 2016; Marrone, 2016; Russell, 2022; Vilas-Boas and Davel, 2018; Windiarti et al., 2014; Yukl, 2013).

Culture involves knowledge sharing and meaning systems among employees (Akiga and Lowe, 2004). Knowledge systems refer to the ways in which knowledge is created, organised, and shared within a given society or culture. They include, for example, the teaching methods and institutions through which knowledge is transmitted, as well as the beliefs, values, and assumptions that shape how knowledge is understood and applied. Meaning systems refer to the ways in which people interpret the world. They include cultural, religious, and philosophical beliefs, values, and assumptions that shape how people interpret and make sense of their experiences and the events that occur around them. Both of these systems shape how individuals and groups understand and interact with the surrounding world. Hence, leaders and followers from different cultural backgrounds with different socialisation norms have different leadership prototypes and, consequently, different expectations regarding leader-follower interaction and expected behaviours (Derungs, 2010; Festing and Maletzky, 2011; Hanges et al., 2016; Karim, 2003; Morrison, 2012; Suutari et al., 2002; Tsai, 2022; Yukl, 2013).

In nursing, transcultural leadership is a consequence of increased cultural diversity in populations in need of care (Marrone, 2016; Russell, 2022), increased cultural diversity in nursing teams, and the need to prepare government officials, practitioners, academic and clinical administrators, teachers, researchers, consultants, minorities, and others for transcultural nursing (Leininger and McFarland, 2002). Transcultural nursing is a field of nursing knowledge that studies differences and similarities in human care, beliefs, values, and standardised lifestyles across cultures, providing nurses with ethical insights into diseases, treatments, and issues related to death in different cultures (Leininger and McFarland, 2002). According to Lowenstein and Glanville (1995, 1991), transcultural nursing knowledge facilitates meeting the needs of culturally-diverse populations, providing culturally-congruent care and mediating conflicts between nurses from different cultural backgrounds. It also prevents cultural pain, shock, conflict, ethnocentrism, and the imposition of work practices that may result in dissatisfaction with the work environment, decreased quality of care, or culturally destructive and unethical care (Leininger and McFarland, 2006).

#### 3.6.2 Consequences

The consequences of transcultural leadership are the positive outcomes that result from the effective implementation of this leadership approach. The key consequences of transcultural leadership identified in the literature are listed in Table 3.

**Table 3:** Consequences of transcultural leadership.

Consequences	
Overall	In nursing
■ Overcoming cultural differences (Derungs, 2010; Foulkes, 1994; Smith, 2013); ■ Inclusion (Smith, 2013); ■ Shared meaning and common purpose (Derungs, 2010); ■ Multicultural synergy (Foulkes, 1994).	■ Culturally-congruent care to patients (Russell, 2022); ■ Optimal health outcomes for all populations (Russell, 2022).

Overall, there are positive consequences for a team, such as synergy and improved performance, after overcoming cultural differences that may lead to conflict. In nursing, transcultural leadership leads particularly to the delivery of culturally-congruent care to patients and optimal health outcomes for all populations (Russell, 2022), which may require interventions by nurse leaders and managers at the organisational, unit, and nursing team levels, as suggested in the literature (Teixeira et al., 2022a).

### 3.7 Empirical referents

Empirical referents demonstrate the occurrence of a concept through its existence or presence (Walker & Avant, 2019). Although Walker and Avant (2019) argue that empirical referents are not tools to measure the concept, several researchers identified tools as possible means to measure and demonstrate the concept under analysis (Mosed et al., 2021; Ntshingila et al., 2021; Sharifi et al., 2019). Through concept analysis, the absence of quantitative measures for transcultural nursing leadership was identified. Considering the increasing cultural diversity among patients and nurses, we argue that a measurement tool must be developed to assess nurses’ transcultural leadership in culturally-diverse nursing work environments; that is, in healthcare settings where nurses from different cultural backgrounds work, and care is provided or can be provided to culturally-diverse populations. This tool may be based on deductive and inductive methods, such as literature reviews and exploratory studies, both possible sources for developing new tools (Polit and Beck, 2021).

### 3.8 Operational definition of transcultural nursing leadership

Based on this concept analysis, we propose defining transcultural nursing leadership as a culturally sensitive transformation journey of behaviours, processes, and products adapted to the cultural needs of nurses and patients, breaking conventional paradigms and guiding the delivery of culturally-congruent care, that results in nursing teams able to overcome cultural differences and work in synergy to accomplish goals, provide culturally-congruent nursing care, and improve optimal health outcomes for all populations.

## 4 Discussion

Transcultural nursing leadership is a complex and evolving concept that plays a critical role in addressing the challenges and opportunities associated with providing culturally-congruent care and leading nurses from different cultural backgrounds in diverse healthcare settings. In this section, we examine the definitions and attributes of transcultural nursing leadership; discuss its conceptual framework; review empirical findings related to this concept; reflect on its conceptual clarity; and explore practical implications, limitations, and future directions for further research and practice.

In this concept analysis, we found that transcultural nursing leadership was a process of leader, nurse, and organisational transformation that meets the needs of nurses and patients from different cultural backgrounds and is conducive to better outcomes for both. Attributes of transcultural nursing leadership may include sensibility to cultural differences and power imbalances; transcultural competence; adaptation of behaviours, processes, and products; breaking conventional paradigms; and a transformation journey that fosters team synergy and optimal patient health outcomes.

The proposed operational definition was partially derived from the definitions and characteristics of transcultural leadership described in fields other than nursing. The nursing literature on transcultural leadership to date has focused primarily on the needs of patients from different cultural backgrounds. The identified attributes, antecedents, and consequences in the nursing literature were mainly related to patients and did not cover the complexity of a culturally-diverse nursing work environment, which includes the challenges faced by those leading nursing staff from different cultural backgrounds. This may indicate that the concept is still maturing and adjusting to the reality of culturally-diverse nursing teams in several countries because of the current international mobility of nurses, which is expected to become an even bigger issue in the near future (Buchan et al., 2022). To fill this gap, the attributes, antecedents, and consequences found in the overall literature on transcultural leadership were considered and added to those from the nursing literature.

In nursing, transcultural leadership seems to be a concept grounded in the Cultural Care Diversity and Universality theory proposed by Leininger, which emphasises the need for nurses to recognise and address the unique cultural values, beliefs, practices, and experiences of individuals and communities (Leininger and McFarland, 2006). Similarly, this theory advocates the necessity for nursing service and education leaders to meet not only the needs of patients but also of students, faculty, and staff from diverse cultures, stating that all ‘expect that nursing administrators and staff will make decisions and judgements that meet or at least consider their cultural needs as human rights’ (Leininger, 2006b, p. 368). It also supports the need to develop ways to use the talent and assets of nurses from different cultures and to maximise and use their knowledge and skills rather than discredit, demean, or misuse nursing talents (Leininger, 2006b, p. 368).

Although Leininger's assumptions support our proposed operational definition of transcultural nursing leadership, ‘administrators’ or ‘administration’ are more frequently used than ‘leaders’ or ‘leadership’. Could we consider the concept of transcultural nursing leadership an evolution or subdivision of transcultural nursing administration as defined by Leininger? McFarland and Wehbe-Alamah (2019) proposed future directions using Culture Care Theory to guide culturally competent administrative and leadership policies and procedures. Therefore, it is our understanding that transcultural nursing leadership and transcultural nursing administration have independent definitions.

Empirical research on transcultural nursing leadership remains limited; however, existing studies highlight the importance of nurse managers’ leadership practices in improving nurses’ and patients’ outcomes and promoting culturally competent nursing work environments (Teixeira et al., 2022b, 2022a).

The concept of transcultural nursing leadership lacks clarity because of the interchangeable use of similar concepts. Several terms have been used in the literature to describe the phenomenon of leadership in culturally-diverse work environments, including cross-cultural, intercultural, transcultural, and global leadership. Based on the CFA, it was observed that there were similarities and differences between them. Transcultural leadership seems distinguished by its inherent ‘transformation journey’ as suggested by Derungs (2010). Further refinement and clarification of this concept may be required to enhance conceptual clarity and facilitate its application in research and practice. Transcultural nursing leadership can only be inferred from its theoretical consequences and can be indirectly measured. Additional research is required to fully understand this concept and develop a robust measurement tool.

We understand that transcultural nursing leadership has significant practical implications for nursing management, education, research, and policy. Findings derived from this concept analysis can help strengthen the questionnaires used to measure leadership in culturally-diverse nursing work environments and assess whether nurse managers’ practices improve patient care and favourable work environments for nurses.

Nurse managers who demonstrate transcultural leadership competencies are crucial in promoting team building, improving performance, creating inclusive work environments, and improving patient outcomes in culturally-diverse healthcare settings. They can also facilitate the development of culturally responsive policies and guidelines, contribute to the design and implementation of culturally-competent nursing education programs, and advocate for integrating transcultural nursing leadership into nursing education. Teaching leadership theory without discussing cultural contexts can create a major gap in preparing professionals to meet the contemporary demands and challenges that leaders face in an increasingly interdependent, diverse, and culturally pluralistic world (Karim, 2003). Therefore, our findings can be integrated into nursing leadership programs to prepare nurses to understand and lead culturally-diverse healthcare settings.

### 4.1 Limitations

There are some limitations to consider when interpreting these results. The search strategies were simple and were not adjusted for each database. Therefore, documents that defined or described the concept of transcultural leadership, which could be important for this concept analysis, may have been missed. Studies of interest may also have been missed due to being published in other languages. However, we believe that searching for documents in English, Spanish, and Portuguese was an asset of this study, as it allowed for a broad spectrum of analysis.

The software interface was defined in Portuguese. Data extracted from papers, books, book chapters, and encyclopaedias written in English were translated into Portuguese to build a text *corpus* for analysis. Therefore, translation limitations and inconsistencies between languages may have influenced the data interpretation.

The operational definition of transcultural nursing leadership may be biased by attributes identified in the literature. However, we understand that, without them, the definition falls far short of the complexity of leadership in culturally-diverse nursing work environments. As this is the first study to delimit the concept of transcultural nursing leadership, further research is needed to refine its definition, attributes, and measurement and to improve its delimitation within the nursing domain.

Empirical research on transcultural nursing leadership needs to be improved, and more studies are needed to explore its impact on patient outcomes, nursing practices, and organizational outcomes. Additionally, the cultural context and diversity of healthcare settings may influence the application of transcultural nursing leadership, and further research is needed to understand how these factors impact its effectiveness.

## 5 Conclusions

A concept analysis of transcultural nursing leadership was conducted using Walker and Avant's method. Transcultural nursing leadership is a concept grounded in nursing theory and has significant implications for nursing management, education, research, and policy. The concept holds great promise for advancing culturally-congruent care, addressing health disparities, and building highly-inclusive and productive nursing teams in an increasingly diverse world. However, further refinement and clarification are required to enhance conceptual clarity and enable its application.

## Funding sources

This study was supported by the Nursing Research Innovation and Development Centre of Lisbon (CIDNUR), Lisbon, Portugal.

## CRediT authorship contribution statement

**Gisela Teixeira:** Conceptualization, Methodology, Software, Data curation, Writing – original draft. **Paulo Cruchinho:** Methodology. **Pedro Lucas:** Writing – review & editing. **Filomena Gaspar:** Supervision.

## Declaration of Competing Interest

The authors declare that there are no conflicts of interest.

## Appendix Supplementary materials

Image, application 1

## References

[bib0001] Abyad A. (2011). Intercultural leadership and communication in global business. Middle East J. Bus..

[bib0002] Akiga, Lowe K.B. (2004). Cross-cultural leadership. Encycl. Leadersh..

[bib0003] Andrews M. (2016).

[bib0004] Bertin M., Atanassova I. (2015). CEUR Workshop Proceedings.

[bib0005] Bird A., Mendenhall M.E. (2016). From cross-cultural management to global leadership: Evolution and adaptation. J. World Bus..

[bib0006] Bonsu S., Twum-Danso E. (2018). Leadership style in the global economy: a focus on cross-cultural and transformational leadership. J. Mark. Manag..

[bib0007] Bragadóttir H., Potter T.M., Pechacek J.M., Bjarnadóttir T. (2020). Transforming global leadership skills in graduate nursing programs using an intercultural setting and a case study on refugees. Interdiscip. J. Partnersh. Stud..

[bib0008] Buchan J., Catton H., Shaffer F. (2022).

[bib0009] Cabrera A., Unruh G. (2012).

[bib0010] Caligiuri P., Tarique I. (2012). Dynamic cross-cultural competencies and global leadership effectiveness. J. World Bus..

[bib0011] Camargo B.V., Justo A.M. (2018). Tutorial para uso do software IRaMuTeQ (Interface de R pour les Analyses Multidimensionnelles de Textes et de Questionnaires). Temas em Psicologia.

[bib0012] Cunha M., Rego A., Cunha R., Cabral-Cardoso C. (2007).

[bib0013] Derungs I., Derungs I. (2010). Trans-Cultural Leadership for Transformation.

[bib0014] Douglas M.K., Rosenkoetter M., Pacquiao D.F., Callister L.C., Hattar-Pollara M., Lauderdale J., Milstead J., Nardi D., Purnell L. (2014). Guidelines for implementing culturally competent nursing care. J. Transcult. Nurs..

[bib0015] Ersoy A. (2014). The role of cultural intelligence in cross-cultural leadership effectiveness: a qualitative study in the hospitality industry. J. Yasar Univ..

[bib0016] Festing M., Maletzky M. (2011). Cross-cultural leadership adjustment — a multilevel framework based on the theory of structuration. Hum. Resour. Manag. Rev..

[bib0017] Foulkes R. (1994). Transcultural leadership: Empowering the diverse workforce. Columbia J. World Bus..

[bib0018] Frawley J., Fasoli L. (2012). Working together: intercultural leadership capabilities for both-ways education. Sch. Leadersh. Manag..

[bib0019] Hajisoteriou C., Angelides P. (2014). Facing the ‘challenge’: school leadership in intercultural schools. Educ. Manag. Adm. Leadersh..

[bib0020] Handin K., Steinwedel J.S. (2006). Developing global leaders: executive coaching targets cross-cultural competencies. Glob. Bus. Organ. Excell..

[bib0021] Hanges P.J., Aiken J.R., Park J., Su J. (2016). Cross-cultural leadership: leading around the world. Curr. Opin. Psychol..

[bib0022] Harvey S. (2015). Transcultural women leaders. SAM Adv. Manag. J..

[bib0023] Karim A.U. (2003). A developmental progression model for intercultural consciousness: a leadership imperative. J. Educ. Bus..

[bib0024] Klamt L., Santos V.dos (2021). The use of the IRAMUTEQ software in content analysis - a comparative study between the ProfEPT course completion works and the program references. Res. Soc. Dev..

[bib0025] Lakshman C. (2013). Biculturalism and attributional complexity: cross-cultural leadership effectiveness. J. Int. Bus. Stud..

[bib0026] Leininger M. (2006).

[bib0027] Leininger M. (2006).

[bib0028] Leininger M. (2002). Transcultural Nursing: Concepts, Theories, Research, and Practice.

[bib0029] Leininger M., McFarland M. (2006).

[bib0030] Leininger M., McFarland M. (2002).

[bib0031] Lowenstein A.J., Glanville C. (1995). Cultural diversity and conflict in the health care workplace. Nurs. Econ..

[bib0032] Lowenstein A.J., Glanville C. (1991). Transcultural concepts applied to nursing administration. J. Nurs. Adm..

[bib0033] Madureira V., da Silva D., Trentini Mercedes, de Souza S. (2021). Conceptual analysis methods in nursing: a theoretical reflection. Esc. Anna Nery.

[bib0034] Marrone S.R. (2016). President's message: transcultural nursing: leadership and innovation. J. Transcult. Nurs..

[bib0035] McFarland M., Wehbe-Alamah H. (2019). Leininger's theory of culture care diversity and universality: an overview with a historical retrospective and a view toward the future. J. Transcult. Nurs..

[bib0036] Morrison J. (2012). A review of “contemporary leadership and intercultural competence: exploring the cross-cultural dynamics within organizations. J. Educ. Bus..

[bib0037] Mosed H., Periord M., Caboral-Stevens M. (2021). A concept analysis of intercultural communication. Nurs. Forum.

[bib0038] Ntshingila N., Downing C., Hastings-Tolsma M. (2021). A concept analysis of self-leadership: the “bleeding edge” in nursing leadership. Nurs. Forum.

[bib0039] Polit D., Beck C. (2021).

[bib0040] Purnell L. (2005). The Purnell model for cultural competence. J. Multicult. Nurs. Heal..

[bib0041] Rao-Nicholson R., Carr C., Smith S. (2020). Cross-cultural leadership adjustment: a strategic analysis of expatriate leadership at a British multinational enterprise. Thunderbird Int. Bus. Rev..

[bib0042] Russell G.P. (2022). President's message: national nurses month-the importance of recognizing transcultural nursing leaders. J. Transcult. Nurs..

[bib0043] Salviati M.E. (2017).

[bib0044] Scandura T., Dorfman P. (2004). Leadership research in an international and cross-cultural context. Leadersh. Q..

[bib0045] Sertel G., Karadag E., Ergin-Kocatürk H. (2022). Effects of leadership on performance: a cross-cultural meta-analysis. Int. J. Cross Cult. Manag..

[bib0046] Sharifi N., Adib-Hajbaghery M., Najafi M. (2019). Cultural competence in nursing: a concept analysis. Int. J. Nurs. Stud..

[bib0047] Smith J.E. (2013).

[bib0048] Story J. (2011). A developmental approach to global leadership. Int. J. Leadersh. Stud..

[bib0049] Suutari V., Raharjo K., Riikkilä T. (2002). The challenge of cross-cultural leadership interaction: Finnish expatriates in Indonesia. Career Dev. Int..

[bib0050] Teixeira G., Gaspar F., Lucas P. (2022). New Trends in Qualitative Research.

[bib0051] Teixeira G., Lucas P., Gaspar F. (2022). International Portuguese nurse leaders’ insights for multicultural nursing. Int. J. Environ. Res. Public Health.

[bib0052] Tsai C.-J. (2022). Cross-cultural leadership behavior adjustment and leader effectiveness: a framework and implications. Int. Stud. Manag. Organ..

[bib0053] Tsai C.-J., Carr C., Qiao K., Supprakit S. (2019). Modes of cross-cultural leadership adjustment: adapting leadership to meet local conditions and/or changing followers to match personal requirements?. Int. J. Hum. Resour. Manag..

[bib0054] Vilas-Boas O., Davel E. (2018). Prática intercultural da liderança: princípios e desafios da pesquisa empírica. Teor. e Prática em Adm..

[bib0055] Walker A., Shuangye C. (2007). Leader authenticity in intercultural school contexts. Educ. Manag. Adm. Leadersh..

[bib0056] Walker L., Avant K. (2019).

[bib0057] Windiarti I.S., Ferris T.L.J., Berryman M.J. (2014). 2014 IEEE International Systems Conference Proceedings, Systems Conference (SysCon), 2014 8th Annual IEEE. IEEE.

[bib0058] Yukl G. (2013).

