# Supplementary material for: Transcultural nursing leadership: A concept analysis
Source: Int J Nurs Stud Adv. 2023 Nov 3;5:100161. doi: 10.1016/j.ijnsa.2023.100161 (PMC11080340; doi:10.1016/j.ijnsa.2023.100161)
Supplement: Supplementary file 1 [file mmc1.docx]

#
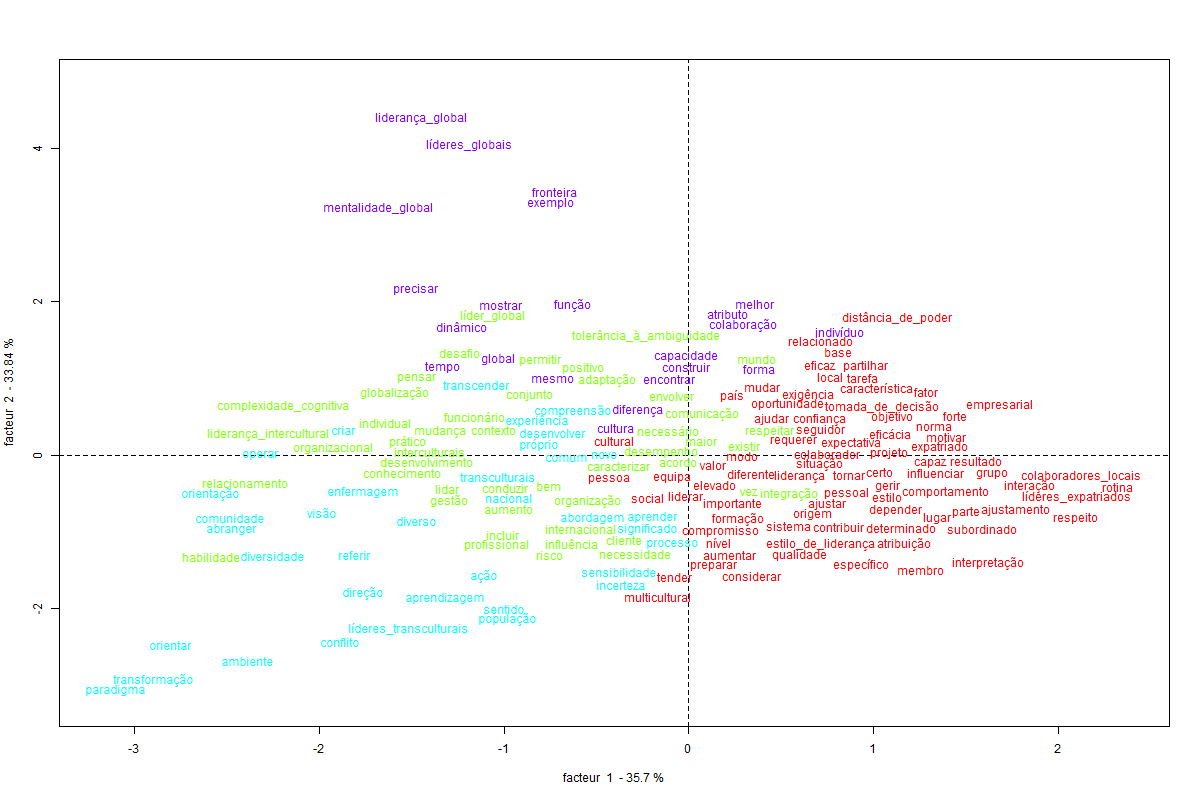
Supplementary Material 1

Correspondence Factor Analysis of the most frequent active forms in the concepts of cross-cultural leadership (red), intercultural leadership (green), transcultural leadership (blue) and global leadership (purple). Sample of the words with the highest occurrence in each concept, with a minimum absolute frequency equal to or higher than five.
